# Treatment escalation for people with anorexia nervosa: setting, therapies and nutritional interventions

**DOI:** 10.1097/YCO.0000000000000964

**Published:** 2024-09-04

**Authors:** Hubertus Himmerich, Johanna Louise Keeler, Kate Tchanturia, Janet Treasure

**Affiliations:** a Centre for Research in Eating and Weight Disorders (CREW), Department of Psychological Medicine, Institute of Psychiatry, Psychology, and Neuroscience, King's College London; b South London and Maudsley NHS Foundation Trust, London, UK

**Keywords:** algorithm, anorexia nervosa, nutrition, setting, therapy, treatment escalation

## Abstract

**Purpose of review:**

Adult patients with severe anorexia nervosa often receive the same unsuccessful treatment without changes regarding the setting, the therapies, or nutritional interventions.

**Recent findings:**

Settings where people with anorexia nervosa are treated include their general practitioner, an independent psychiatric practice, a community mental health team (CMHT), a specialized eating disorder outpatient service, eating disorder early intervention services, a highly intensive eating disorder outpatient or home treatment programme, eating disorder daycare, an inpatient eating disorder service, a general hospital or a general psychiatric hospital, or residential treatment. At a specialized eating disorder service, patients should be offered evidence-based psychotherapy for anorexia nervosa, dietary advice and physical health monitoring as a first step. Additionally, they may be allocated to a specific treatment pathway, family interventions and creative therapies. As a second step, clinicians may consider integrating interventions targeting psychiatric or physical comorbidities, medication for anorexia nervosa or noninvasive neurostimulation. After several years of futile treatment, deep brain stimulation (DBS) should be considered to prevent a chronic course of anorexia nervosa. Nutritional interventions can be escalated from nutritional counselling to nasogastric tube feeding. Patients who rely on nasogastric tube feeding might benefit from percutaneous endoscopic gastrostomy (PEG). Patients who vomit despite a nasogastric tube, might need nasojejunal tube feeding.

**Summary:**

Treatment for people with anorexia nervosa should be regularly reviewed and, if necessary, escalated to avoid a chronic and longstanding disease course.

## INTRODUCTION

### Purpose of the review

The concepts of anorexia nervosa and its treatment have substantially changed as psychological therapies such as the Maudsley Model of Anorexia Nervosa Treatment for Adults (MANTRA), Cognitive Behaviour Therapy for Eating Disorders (CBT-E) and Focal Psychodynamic Therapy for Anorexia Nervosa (FPT) have been found effective for most patients with anorexia nervosa [1–3], especially those in the early stages. However, these therapies have limited success in severely affected people or people who have been suffering from anorexia nervosa for many years.

Despite the availability of the evidence-based psychological treatments, a cohort study in people with eating disorders showed that only ∼30% of patients with anorexia nervosa recovered after 9 years, and only ∼60% after 22 years [4]. Thus, there have even been suggestions out of therapeutic desperation that patients with anorexia nervosa who become chronically ill and do not see a path out of their treatment journey should be offered palliative care and that medical aid in dying should be included in the jurisdictions where such care is legal [5]. However, biological therapeutic options like medication, and emerging options such as noninvasive neuromodulation or deep brain stimulation (DBS), are not often utilized, even though there is some evidence for neuromodulation as an escalatory therapy option for those who do not respond to standard therapy [6^▪^,^7^].

In principle, there are two main approaches to improve treatment success: One is to optimize the treatment for the individual patient, the other option is to escalate the treatment by using a more effective therapy.

In this article, we propose a structured approach to avoid treatment resistance and improve the results of anorexia nervosa treatment, which combines tailored treatment and the treatment escalation approach by focussing on the setting, the treatment and nutritional therapy. We reflect that we are describing a clinical vision and not evidence-based guidance for therapeutic decisions as some of the treatment options are currently under development, and defined treatment sequences have not yet been tested in anorexia nervosa. 

**Box 1:**
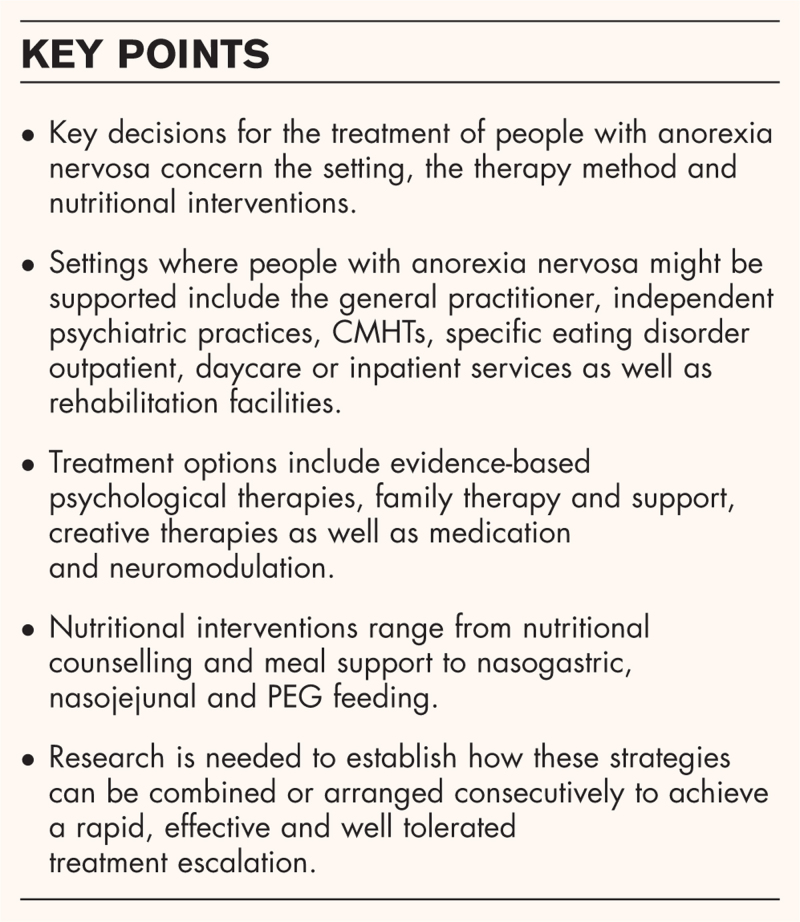
no caption available

### Tailored treatment pathways

Treatment can be individually tailored according to the duration of the illness, comorbidities, symptom clusters or biomarkers. Examples of tailored eating disorder pathways are the First episode and Rapid Early intervention for Eating Disorders (FREED) pathway [8], the Pathway for Eating disorders and Autism developed from Clinical Experience (PEACE) [9] or the pathway for people with type 1 diabetes and disordered eating (T1DE) [10].

Tailored treatments [11] and algorithms to escalate treatment [12] have been developed for people with depression, which is highly comorbid with anorexia nervosa and has a negative influence on weight recovery [13]. Regarding individually tailored treatment, they have suggested the application of subtypes characterized by biomarkers of depression. Therefore, they have postulated two different subtypes of depression according to symptom clusters: type 1, which is characterized by loss of appetite and body weight, insomnia, and suicidal ideation, and type 2, also known as atypical depression, which presents with increased appetite and weight gain, leaden paralysis, hypersomnia and a persistently poor metabolic profile [14].

In order to tailor treatments, biomarkers that predict treatment outcome include electroencephalography (EEG)-measured brain arousal regulation, brain structural or functional findings, genomic, proteomic, and metabolomic markers [15,16]. In anorexia nervosa, however, such subclassifications and individualized predictors of treatment response have not yet been established.

### Treatment escalation

For the treatment of depressive disorders, clinical researchers have also developed an algorithmic approach to improve treatment success: If a patient does not respond to standard therapy with second-generation antidepressants such as selective serotonin reuptake inhibitors (SSRIs), then lithium augmentation, switch of the antidepressant medication to monoamine oxidase inhibitors (MAO-I) or electroconvulsive therapy will follow at specified time points [12,17]. However, so far, no treatment algorithm to escalate the therapeutic intensity has been developed for people with anorexia nervosa.

In anorexia nervosa, novel psychological and biological therapies that could be added to standard therapies in order to escalate or augment treatment have been suggested, developed and tested. New psychological options include Cognitive Remediation Therapy (CRT) [18], cognitive remediation and emotional skills training (CREST) [19], narrative therapies [20,21], forms of exposure therapy such as virtual reality-supported exposure therapy [22], cognitive bias modification training [23], attention bias modification training (ABMT) [24] and avatar-supported therapy [25] which are currently in the early stages of development. Additionally, therapies to educate, support and include the family into the treatment of a person with anorexia nervosa are available and have been proven to be successful [26].

Current and potential future treatment options include neuromodulation and novel psychopharmacological approaches like metreleptin, psilocybin and ketamine and microbiome-based interventions [27]. Thus, it is timely to think about strategies that involve a chain of different treatments for people who do not respond to the first-line treatment instead of offering only one established psychotherapy within an eating disorder service regardless of whether a patient responds to this treatment or not.

## SETTING

### General practitioners

The general practitioner is often the first point of contact for a person with anorexia nervosa. The general practitioner will assess physical and mental health and might offer to refer a patient with anorexia nervosa to a specialist eating disorder service. A general practitioner can also monitor a patient's physical health and offer basic psychotherapy. They might refer a patient to a general adult psychiatrist, psychotherapist, or a community mental health team (CMHT), particularly in areas where no specialist eating disorder service is available. In cooperation with an eating disorder service, a general practitioner might even continue to monitor physical health parameters and prescribe medication for the eating disorder or for comorbid physical or mental health issues. Thus, general practitioners play a key role in steering a person's journey to recovery as reducing the length of untreated illness is a key factor in outcome [8].

### Specialist eating disorder services

Depending on their clinical experience with eating disorder patients, an independent psychiatrist, psychotherapist or a member of a CMHT might refer a patient with anorexia nervosa to a specialized eating disorder service. Here, the multidisciplinary team (MDT) will make decisions on whether they accept the patient in their service or whether they refer the person to more intensive forms of treatment, for example, inpatient, daycare or home treatment settings. The first level may be an offer of weekly psychotherapy as an outpatient, physical health monitoring, dietetic counselling and support and advice for the carers. The MDT might also allocate a patient to a specific pathway, for example, the FREED [8], the PEACE [9], or the T1DE pathway [10].

Evidence has been generated for each of these pathways. The FREED pathway, for example, has been shown to be associated with reduced duration of untreated anorexia nervosa, improved clinical outcomes and increased acceptance and understanding of difficulties by patient [28]; and the benefit of early intervention has been recognized [8].

### Inpatient hospital treatment

Inpatient treatment within general hospitals may be needed for acute physical health conditions, for example, acute severe electrolyte disturbances. Treatment on a general psychiatric ward may be needed if a patient suffers from a comorbid mental health disorder such as emotional unstable personality disorder with suicidal tendencies. If a patient needs more time to weight-restore or needs social rehabilitation, an eating disorder-specific rehabilitation unit or even a general rehabilitation unit might be considered.

Table 1 summarizes the treatment settings and outlines the criteria that are indicative of the respective setting.

**Table 1:** Treatment settings for people with anorexia nervosa and corresponding decision criteria

Treatment setting	Decision criteria indicative of this setting
GP	First contact Differential diagnosis, exclusion of physical health cause General physical health monitoring Continuous prescriptions (medicine, physiotherapy, social prescribing)
Eating disorder outpatient service	Specific eating disorder diagnosis Eating disorder physical and mental health monitoring Need for specific eating disorder psychotherapy and dietary advice Patient physically and mentally stable
Independent psychiatrist or psychotherapist	Psychotherapy or psychiatric treatment only No multidisciplinary team input needed
CMHT	Psychiatric comorbidities (obsessive–compulsive disorder, personality disorders) Risk of mental health crisis or suicidality Social care part of treatment plan
Intensive outpatient service or day care	Outpatient service not sufficient Inpatient treatment to be avoided. Transition from outpatient to inpatient treatment and back
Inpatient eating disorder treatment	Unstable physical health Severe weight loss in the community Use of Mental Health Act (or equivalent) Nasogastric tube feeding 1 : 1 or continuous observation necessary
General psychiatric hospital	Severe mental health comorbidities that cannot be managed by community mental health team Acute suicidality
General hospital	Severe physical health comorbidities
Residential treatment	Transition from inpatient to outpatient treatment Necessity to promote independence Weight recovery slow during inpatient treatment Occupational and spare time rehabilitation

CMHT, community mental health team; GP, general practitioner.

The literature on how to map a treatment escalation pathway for the treatment of anorexia nervosa is scarce. For a review of settings with a high level of care, see [29,30].

Two recent studies compared inpatient treatment and partial hospital treatment. Herpertz-Dahlmann *et al.*[31] compared daycare treatment after short inpatient care versus continued inpatient treatment in adolescents with anorexia nervosa in a multicentre, randomized, open-label, noninferiority trial. They found that the average length of stay in both the inpatient arm and the day care arm were 3–4 months. However, no differences in outcome between day care and inpatient was found. Short inpatient treatment plus day care treatment was equally safe and less costly compared to full inpatient treatment [31].

Another study, that aimed to test the clinical effectiveness and cost-effectiveness of a 'stepping into day treatment’ approach versus inpatient treatment as usual for anorexia nervosa in adult specialist eating disorder services [32] failed to recruit a sufficient number of patients. Fifteen patients with anorexia nervosa (of 53 approached) participated in the study and were followed up to 6 or 12 months. At baseline, participants in both trial arms rated stepped-care daycare treatment as more acceptable. At 12 months, participants’ BMIs had increased in both trial arms. No conclusions could be drawn concerning the effectiveness of inpatient and stepped-care day patient treatment, but the latter was perceived more positively [33]. Patient-related, service-related and systemic factors such as the COVID-19 pandemic contributed to the trial's failure. However, it also demonstrates how difficult it is to perform randomized controlled trials (RCTs) that compare different settings because of the patients’ and the clinicians’ strong opinion about the most desirable treatment and the fact that severely affected patients or patients who lack insight are not deemed safe to be treated in outpatients from the start.

People with anorexia nervosa should only be admitted to an inpatient service for medical stabilization or to initiate refeeding, if their physical health is severely compromised [34,35]. When deciding whether day patient or inpatient care is most appropriate, the BMI or weight, and also the rate of weight loss (>1 kg/week) should be taken into account, as well as the need to actively monitor medical risk parameters [blood tests, physical observations and electrocardiography (ECG) for bradycardia or prolonged QT interval], the person's current overall physical health and whether carers can support them and keep them from significant harm as an outpatient or day patient. Inpatient care should not solely be used to provide psychological treatment [34]. Equally, patients with anorexia nervosa should not be discharged just because they have reached a healthy weight [34]. For patients with acute mental health risk, a psychiatric crisis team which might be part of a community mental health team, or a general psychiatric inpatient setting might be more appropriate. Ideally, an inpatient treatment should be brief to stabilize a patient's physical health. However, clinical reality and audit data suggests that hospital stay is often longer than a few weeks. In fact, the average length of stay for inpatients in the UK is ∼130 days [36].

## PSYCHIATRIC AND PSYCHOTHERAPEUTIC TREATMENT

Any anorexia nervosa treatment should include regular shared physical health monitoring, psychological therapy and nutritional support, the treatment of comorbidities and family skill sharing [34]. Physical health monitoring consists of regular physical examinations including the measurement of the correct body weight [37], heart rate, body temperature, muscle strength, the determination of electrolyte levels, liver and renal function tests and leukocyte levels, as well as ECG, to increase the short-term safety of a patient, whereas a dual X-ray absorptiometry (DEXA) scan helps to assess the long-term consequences of osteoporosis [35,38].

In clinical practice, creative therapies are usually offered if available and applicable, and are usually mentioned in local or national treatment standards, e.g., [39,40]. On top of these basic elements, the treatment can by escalated by adding a medication or neuromodulation. Table 2 details information about the available treatments and the decision criteria indicative of the respective treatment.

**Table 2:** Psychiatric and psychological treatments for people with anorexia nervosa and corresponding decision criteria

Treatment	Decision criteria indicative of this treatment
Physical health monitoring	Recommended regularly for every patient with anorexia nervosa Frequency should increase during weight loss or refeeding Necessity to monitor harmful behaviours, for example, vomiting and laxatives abuse Should take physical comorbidities such as osteoporosis and type 1 diabetes into account
Psychological therapy	Recommended for every patient with anorexia nervosa Type of therapy depends on: Motivation and ability of the patient Comorbidities (e.g. autistic traits, type 1 diabetes) Training and experience of the therapist Availability Technical requirements
Family-based interventions	Recommended regularly for every patient with anorexia nervosa Consent of adult patients necessary Type of family-based intervention: Training and experience of the therapist Motivation and availability of the carers
Creative therapies	Should be offered to all patients with anorexia nervosa
Treatment of comorbidities	Recommended for all patients with anorexia nervosa
Emerging interventional approaches	
Medication	Nonresponse to usual treatment Shared decision to try a medication Specific effects of the medication help with corresponding symptoms, for example Olanzapine and dronabinol to increase weight Metreleptin to reduce physical activity Ketamine to help with depression
Noninvasive neuromodulation, for example, repetitive transcranial magnetic stimulation (rTMS) or transcranial direct current stimulation (tDCS)	rTMS or tDCS available Nonresponse to usual treatment Shared decision to try rTMS or tDCS
Invasive neuromodulation, for example, deep brain stimulation	Treatment-resistant anorexia nervosa All other treatment options exhausted Psychiatrist and neurosurgeon confirm indication Patient consents to deep brain stimulation

### Psychological interventions

#### Motivational strategies

At the beginning and throughout the psychological support for a person with anorexia nervosa, motivation is a key factor for successful treatment. However, anorexia nervosa might serve a psychological purpose for the affected individual which contributes to their ambivalence towards anorexia nervosa. Therefore, people with anorexia nervosa might have ambivalent feelings towards change. This ambivalence poses a major hurdle within the treatment process [41]. Ambivalence manifests as an unstable readiness to change which, in turn, has been suggested as an important factor in achieving a good treatment outcome [42]. Motivational interviewing and motivational enhancement therapy (MET) have, therefore, been developed to improve a person with anorexia nervosa's motivation to change. However, a recent meta-analysis found no statistically significant effect of MET or motivational interviewing on motivation in people with eating disorders [43]. The authors of this meta-analysis stated that the effect of MET or motivational interviewing on motivation for behavioural change, eating disorder psychopathology and BMI is still unclear, because the individual studies differed substantially in design, and the outcomes were inconsistently assessed with regards to instruments and duration of the intervention. Despite the finding of a lack of evidence for motivational interventions, we assume that strategies of motivational interviewing are nonetheless applied in the clinical care of people with anorexia nervosa as establishing a consistent motivation for change is a prerequisite to initiate anorexia nervosa therapy in a meaningful way [44].

#### Maudsley Model of Anorexia Nervosa Treatment for Adults

MANTRA and CBT-E are the most established psychological therapies for anorexia nervosa [34]. MANTRA is a flexible, identity-based treatment that is usually delivered over 10–20 sessions. It is based on the cognitive-interpersonal model [45] and aims to target the maintaining factors of anorexia, for example, unhelpful thinking styles, including rigidity, perfectionism, and obsessive–compulsive traits, faulty cognition and beliefs, for example, the benefits of anorexia nervosa, emotional avoidance and responses from others that do not support recovery such as criticism or enabling of behaviours [45,46]. MANTRA is specifically designed for anorexia nervosa treatment and is tailored to suit the common temperamental traits associated with the illness to focus on developing a recovery identity, and is delivered using elements of motivational interviewing, emotion-focused therapy and CBT [1,47].

In a number of RCTs comparing treatment for anorexia nervosa, MANTRA was found to have positive outcomes regarding BMI and eating disorder psychopathology; MANTRA was favourably rated by patients and resulted in increasing weight even in severely unwell patients [1,46]. A review of evidence from RCTs comparing treatments for anorexia concluded that MANTRA has a moderate evidence base, which shows that it produces a moderate and lasting beneficial effect [47,48].

#### Cognitive Behaviour Therapy for Eating Disorders

CBT-E is an enhanced version of CBT for eating disorders. It is designed as individual psychological therapy consisting of up to 40 sessions over 20 weeks. CBT-E has a focused version, which is shorter and focussed on eating disorder symptoms and behaviours, and a broad version, which also addresses maintaining mechanisms, such as perfectionism, low self-esteem and interpersonal difficulties. CBT-E aims to alter faulty cognitions by focusing on behavioural changes [49]. There is weak to moderate evidence for CBT-E for adults [47,50]. However, it is unclear whether CBT-E is superior to comparable psychotherapies [51,52]. In an RCT that compared CBT-E, FPT and optimized treatment as usual, BMI increased in all three study groups, and no differences were noted between groups in terms of recovery at 12-month follow-up. FPT proved advantageous in terms of recovery at 12-month follow-up, but CBT-E was more effective with respect to speed of weight gain and improvements in eating disorder psychopathology [53].

#### Cognitive Remediation Therapy and Cognitive Remediation and Emotion Skills Training

MANTRA, CBT-E and FPT require the person with anorexia nervosa to have a certain degree of cognitive flexibility and bigger picture thinking. As people with anorexia nervosa might have difficulties in these domains, particularly at a very low body weight [54,55], CRT [18,56] and Cognitive Remediation and Emotion Skills Training (CREST) have been tested in this patient group [57,58]. Adjunct therapies such as brief individual CRT and CREST are designed to support nutritionally compromised patients firstly to establish a therapeutic alliance, and secondly to explore thinking styles and emotion-regulation strategies and engage experientially in talking therapies.

CRT was originally developed for the rehabilitation of individuals with neuropsychological problems. However, it has been adapted to address the common problem of cognitive inflexibility in people with anorexia nervosa, such as poor set shifting, weak central coherence (bigger picture thinking) and perfectionism. CRT helps to identify thinking strategies using cognitive exercises, in practising switching between tasks, multitasking and bigger picture thinking to break inflexible thinking patterns. CRT can be delivered either with 10 individual 45 min sessions, or as a briefer format in a group setting over five or six sessions. It can be used for patients with very low BMI, unlike most talking therapies, allowing them to engage in psychological work early on in treatment [59]. CRT can be a useful step to begin psychological interventions, as it improves motivation, the therapeutic alliance, cognitive processes and insight/reflection on one's own cognitive strategies. However, CRT is not a stand-alone treatment for eating disorders and does not directly target weight change [46]. CREST is an intervention developed to address problems with identifying, managing and expressing emotions among individuals with anorexia nervosa. Like CRT, it is an intervention that can be offered early on in admission when patients may not benefit from more complex psychological therapies. CREST is generally delivered over 8–10 sessions. Typically, if a patient has previously had CRT, they are offered eight individual sessions of CREST. If patients have not had any experience of CRT, they will first have two sessions focused on thinking styles, followed by eight sessions involving the psychoeducation and experiential elements of CREST [46,57,60]. Detailed studies using qualitative data and self-report questionnaires offer positive feedback and show promise; however, more studies with RCT methodology are required to endorse CREST as a standard adjunct treatment for anorexia nervosa.

#### Emerging psychological therapies

Experimental therapies that are currently being tested for their use in clinical practise for anorexia nervosa treatment include cognitive bias modification training, ABMT and AVATAR therapy [22–25,61]. Virtual reality exposure therapy can, for example, make use of a virtual reality kitchen where a patient can interact with feared foods [22].

ABMT addresses the tendency of people with anorexia nervosa to focus their attention on specific weight-related body parts and on specific foods; and is supposed to increase general attention and promote stimulus re-evaluation [23,24]. Eye-tracking computer tasks, virtual reality paradigms and combinations of the two are currently being tested [61].

#### Group therapies

It is important to highlight the value of group-format psychological therapies. A symptom-based group programme was developed and evaluated to support patients with anorexia nervosa to tolerate the group setting and manage their main symptoms. This group programme is tailored specifically to inpatient admissions. All psychological groups have a brief format and combine psychoeducation with practical experiential exercises [62]. Additional innovative group therapies that might be beneficial particularly for inpatients include the narrative group therapy approach informed by the ‘Tree of Life’ model [63] and sensory wellbeing workshops [64].

### Family and carer interventions

Working with the family can include sharing information with carers, providing guidance and support or facilitating family therapy in the narrow sense that includes full participation of the whole family. Family therapy may be particularly relevant in children and young people with anorexia nervosa.

Families and friends are often motivated to help a loved one with an eating disorder. However, they may be uncertain about how to do it, particularly if they care about an affected adult person. The cognitive interpersonal model of eating disorders describes social interactions that can act as perpetuating factors of anorexia nervosa. These factors are the targets of skill-sharing interventions such as the New Maudsley Collaborative Care approach [65]. Starvation impairs aspects of social cognition, and this can disrupt relationships. In addition, the visible starvation consequences of the eating disorder may cause supporters to become distressed and anxious and either accommodate to the eating disorder or resist with frustration and criticism. Providing supporters with skills to manage their own reactions to these secondary manifestations of severe underweight produces positive benefits for themselves and those they care for and can reduce the need for high-intensity services. Skill-sharing interventions can be delivered as an independent resource for supporters or as a form of augmentation to individual therapy [66].

### Creative interventions

To date, it is unclear whether art has any inherent curative potential; it is rather used as a therapeutic medium to support intrapsychic and interpersonal transformations. People with anorexia nervosa might benefit from the creation of art, the therapeutic relationship with the therapist and the group of participants [67]. Arts and music therapy have been shown to improve the outcome of traditional psychological therapies. For example, music and art therapy were reported to effectively improve patients’ recognition and acceptance of CBT treatment and its therapeutic effects. Creative approaches can also help with adverse emotional reactions (e.g. depression and anxiety) to therapy [68].

Traditional treatment for anorexia nervosa usually focuses on speech-based psychological therapies such as MANTRA or CBT-E. Yet, as a nonverbally mediated therapy, music therapy can find a legitimate place and complement the psychiatric, psychotherapeutic, somatic, dietetic and nursing areas where speech already circulates [69]. Music-based interventions for anorexia nervosa vary widely. In the current literature, they are reported to be associated with improved mood regulation, emotional well being and the management of meal-related distress [70]. A systematic review of music interventions for people with anorexia nervosa showed that listening to classical music was beneficial to food consumption and singing in a group reduced postprandial anxiety in anorexia nervosa inpatients and outpatients [71].

### Medication

#### Olanzapine

Several RCTs [72–76] have been conducted to test the atypical antipsychotic olanzapine for anorexia nervosa in addition to treatment as usual, with four of them finding that olanzapine leads to moderate weight gain in patients with anorexia nervosa [72–75]. However, olanzapine does not consistently show beneficial effects on psychological symptoms including eating disorder psychopathology, depression and anxiety. Due to the reluctance of patients to take olanzapine [72], the low adherence rates [73], the moderate acceptability and reports of either hyperglycaemia or hypoglycaemia in some cases, the World Federation of Societies of Biological Psychiatry (WFSBP) task force for eating disorders gave only a limited recommendation for olanzapine in adult patients in their most recent guidelines on the pharmacological treatment of eating disorders [77^▪▪^].

#### Dronabinol

Andries *et al.*[78,79] conducted a double-blind placebo-controlled crossover study of dronabinol, a form of synthetic tetrahydrocannabinol, in 25 adult patients who had anorexia nervosa for at least 5 years. During the 4 weeks of drug therapy, there was a significant increase in weight gain compared with placebo, but no difference reported in Eating Disorder Inventory (EDI) scores. However, in a 4-week, double-blind crossover trial, Gross *et al.*[80] found that three patients experienced dysphoric reactions, and they did not find differences in weight between dronabinol and diazepam. As the two RCTs showed contradictory results, the WFSBP task force for eating disorders gave only a limited recommendation for the use of dronabinol [77^▪▪^].

#### Metreleptin

Gradl-Dietsch *et al.*[81] described the treatment of a 15-year-old female patient with anorexia nervosa with metreleptin, a human recombinant leptin, for 9 days. The treatment was associated with a self-reported increase in appetite resulting in rapid weight gain, and a substantial improvement of eating disorder cognitions and depressive symptoms. Antel *et al.*[82] reported the case of a 15-year-old adolescent male patient with severe anorexia nervosa with marked hyperactivity who was treated with metreleptin over 9 days. Substantial improvements of mood and eating disorder-related cognitions and hyperactivity started after 2 days of treatment, sub-physiological testosterone and triiodothyronine levels normalized, and weight increased in the follow-up period. Milos *et al.*[83] published a case series of two adult and one adolescent patients with anorexia nervosa. Two of three patients gained weight in the treatment period. They also experienced an improvement in overactivity, repetitive thoughts of food, inner restlessness, fear of weight gain and depression. Therefore, there is some preliminary evidence that metreleptin might be effective in anorexia nervosa [77^▪▪^].

#### Classic and nonclassic psychedelics

Psilocybin is a molecule produced by certain fungi that results in euphoria, changes in consciousness, time distortions, changes in perception and spiritual experiences. A meta-analysis demonstrated the effectiveness of psilocybin in significantly reducing anxiety and depressive symptoms in people with anxiety and depressive disorders [84]. It improves serotoninergic transmission, neuroplasticity, connectivity within the brain, and the production of brain-derived neurotrophic factor and glial cell-derived neurotrophic factor, thus potentially leading to increased cognitive flexibility. An open-label feasibility study in 10 adults with anorexia nervosa demonstrated the safety, tolerability and acceptability of 25 mg psilocybin [85^▪^].

Another promising drug for patients with anorexia nervosa and depression is ketamine. Ketamine is an N-methyl-D-aspartate (NMDA) receptor antagonist, which paradoxically increases glutamate transmission in subanaesthetic doses. It has a rapid but temporary antidepressant effect in people with depression and may alleviate neuroplastic deficits in anorexia nervosa [86^▪^]. Small studies have shown preliminary evidence in people with anorexia nervosa [86^▪^,^87^]. However, no RCTs have been conducted in anorexia nervosa to date. Therefore, feasibility studies investigating the acceptability, safety and tolerability of ketamine in people with anorexia nervosa are needed as a first step towards an RCT.

### Neuromodulation

Neuromodulation could be considered as an alternative to medication in addition to treatment as usual. For the noninvasive approaches, transcranial direct current stimulation (tDCS) and repetitive transcranial magnetic stimulation (rTMS), preliminary results are promising [6^▪^,^7^,^88^,^89^], and there is some evidence for the effectiveness of DBS in treatment-resistant anorexia nervosa [6^▪^,^7^].

## NUTRITIONAL INTERVENTIONS

In people with eating disorders, the establishment of a regular meal pattern is crucial. In patients with anorexia nervosa, weight recovery is a necessary additional meal-related therapeutic goal. Severely affected patients with anorexia nervosa might start with psychotherapeutic work on eating behaviour, which includes meal planning, keeping and evaluating a food diary, increasing the variety of food and reviewing and evaluating progress [49]. Food exposure is a psychotherapeutic approach that can aid nutritional recovery. It can be facilitated *in vivo* but as aforementioned is also being tested within a virtual reality paradigm [22].

However, severely affected patients usually need inpatient treatment where intensive professional meal support by a health professional sitting alongside the patients on the table during mealtimes will be provided. If the patient does not manage to eat with the standard inpatient meal support, which includes the observation of eating behaviour and addressing unhelpful behaviours, support and encouragement at the dining table and direct feedback, nasogastric tube feeding might become necessary [90]. Nasogastric tube feeding has been shown to promote short-term weight gain in people with anorexia nervosa [91].

Nasogastric tube feeding means the insertion of a nasogastric tube and the administration of food through this tube. If a patient does not agree with this refeeding measure, it might be necessary to apply mental health legislation which differs between countries [92]. If a patient becomes reliant on nasogastric tube feeding, and unsuccessful attempts to remove the nasogastric tube have been made, a percutaneous endoscopic gastrostomy (PEG) could be considered as PEG feeding has been reported to be a long-term strategy for selected people with anorexia nervosa who rely on enteral feeding [93]. Nasogastric tube feeding is not a guarantee for refeeding, because a patient can still vomit even with a nasogastric tube or a PEG. Thus, a nasojejunal tube might be considered. However, nasojejunal feeding is a theoretical option without evidence. Table 3 details the options for nutritional interventions and the criteria for increasing nutritional support.

**Table 3:** Nutritional interventions

Nutritional interventions	Decision criteria indicative of this intervention
Nutritional advice and dietetic counselling	Dietetic advice should be available during eating disorder therapy
Psychotherapeutic work on eating behaviour	Outpatient therapy to improve weight recovery
Meal support	Direct in-person meal support Day care or inpatient setting
Nasogastric tube feeding	Weight loss despite inpatient treatment Nasogastric tube feeding technically available Clinical team trained in NG tube feeding
Nasojejunal feeding	Nasogastric feeding contra-indicated Patient vomits despite nasogastric tube feeding and intensive support
Percutaneous endoscopic gastrostomy feeding	Nasogastric tube contra-indicated or not tolerated

NS, nasogastric.

## CONCLUSION

In summary, we have outlined a strategy consisting of three components to escalate the treatment for anorexia nervosa. These components are the setting, the psychiatric and psychotherapeutic treatment, and the nutritional support. The intensity of the setting can be increased by referring the person with anorexia nervosa from the care of a general practitioner, an independent psychiatric practice or a CMHT to a specialized eating disorder service, where they can escalate from outpatient treatment or daycare to inpatient treatment. Alternative hospital treatments take place in general hospitals or general psychiatric hospitals. Residential treatment might be considered after or instead of inpatient eating disorder treatment. The psychiatric and psychotherapeutic treatment should consist of an evidence-based anorexia nervosa psychotherapy, family-based interventions and creative therapies and can be escalated using medication, or in the future with more evidence, potentially experimental or psychedelic-assisted psychotherapy, or neurostimulation. Nutritional interventions range from nutritional counselling and meal support to assisted feeding, nasogastric, PEG and nasojejunal tube feeding.

**FIGURE 1:**
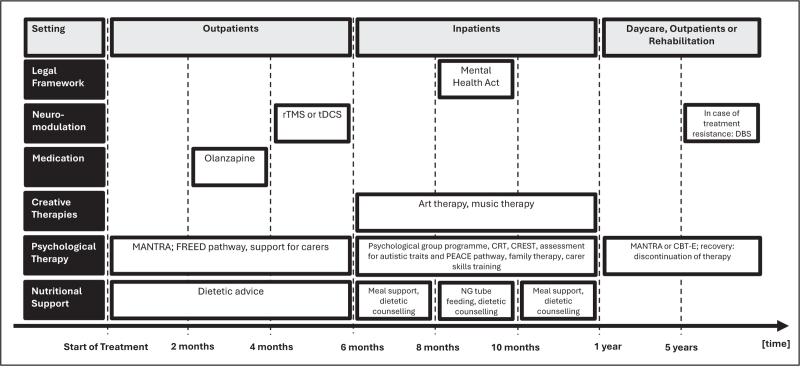
Example of a treatment algorithm for a young person with anorexia nervosa.

To visualize how such a sequence of escalating treatments might look like, we have created an example of a potential treatment algorithm for a young person with the first episode of anorexia nervosa (see Fig. 1).

After a referral from their general practitioner, this young adult person would start as an outpatient in the FREED pathway [28] and receive MANTRA [46] and nutritional counselling. Depending on the patient's preferences and agreement, they might also receive family therapy and/or carer skills training or support [94]. If no treatment success is achieved after 2 months, olanzapine could be added as medication to aid weight recovery [77^▪▪^]. Should no therapeutic breakthrough be achieved after even 4 months of treatment, a form of noninvasive neuromodulation such as tDCS or rTMS could follow [6^▪^,^7^]. After 6 months, the outpatient treatment should be reviewed. If the patient did not manage to gain or even lost weight or showed increased anorexia nervosa symptoms, inpatient treatment might be considered. Inpatient treatment would offer the opportunity for more intensive nutritional support, adjunct therapies (CRT, CREST, brief group programme) [60,62], creative therapies and continued support for carers and family therapy. If necessary and no other option is possible, the Mental Health Act and NG tube feeding can follow to intensify the treatment further [91]. If after 1 year, no breakthrough is made, referral to a rehabilitation unit might be considered. After 5 years of futile treatment, DBS might be considered to be an appropriate measure [7].

However, this suggested algorithm is a proposal, not an evidence-based sequence of treatments. The chosen treatments, the time points to review treatment progress and the criteria for treatment success need to be evaluated. Insights and formulations to facilitate individually tailored therapy might also shape this approach as psychological features, including cognitive testing or biomarkers could help to individualize the algorithm.

In depression, a fixed treatment algorithm for every patient in the participating services was developed. This strict sequential application of treatments which was decided in advance and applicable to every patient with depression improved success rates and reduced direct treatment costs in severely affected depressed patients [12]. This algorithm included critical decision points at the end of each treatment step based on standardized and systematic measurements of response and an algorithm-guided decision-making process [12]. It is unclear whether patients with anorexia nervosa and eating disorder therapists would agree to have their therapeutic freedom and individualized decision-making severely restricted.

Currently, such an approach cannot be translated directly into anorexia nervosa treatment because there are no pharmacological treatments approved or fully recommended [77^▪▪^]. The same applies to noninvasive and invasive neuromodulation. Thus, as there are no approved biological treatments, shared decision-making must play a greater role for therapeutic decisions in anorexia nervosa [95].

To develop an evidence-based treatment algorithm for people with anorexia nervosa in the future, research is needed that compares interventions, informs about the effectiveness of treatments in different treatment settings and the effectiveness of combinations of therapies, as well as their impact from a health economic, social and quality-of-life perspective. However, previous meta-analyses of pharmacological and psychological treatments for outpatient treatment of adult anorexia nervosa have proved to be inconclusive. Network meta-analysis (NMA) has the potential to overcome the limitations of pairwise meta-analysis, as this approach can compare multiple treatments using both direct comparisons of interventions within RCTs and indirect comparisons across trials based on a common comparator [96]. This could be applied across treatment types (i.e. including psychotherapeutic, psychopharmacological, neuromodulation, nutritional). Furthermore, as treatment in anorexia nervosa often fails, clinical research is much needed about what to try next if the first treatment fails.

This review has its limitations because it is not based on a systematic review. There is also variable preliminary evidence for the feasibility and effectiveness of the various forms of novel interventions mentioned (e.g., novel medications, neuromodulation and psychotherapeutic approaches). Additionally, the list of potential new approaches is not exhaustive; there are other promising medications (e.g. mirtazapine or aripiprazole [77^▪▪^]) and psychotherapies (e.g. psychological group therapies to focus on positive emotions [97], positive communications [98] and wellbeing [99]) that are not mentioned or explained. Additionally, the treatment of comorbid mental health problems [100], for example, anxiety and mood disorders, obsessive–compulsive disorder (OCD), autism spectrum disorder (ASD) or emotionally unstable personality disorder, is not sufficiently covered but might also play a significant role in overcoming treatment resistance and escalating the therapeutic strategy for a person with anorexia nervosa.

## Acknowledgements

*None.*

### Financial support and sponsorship

*H.H., K.T. and J.T. have received salary support from the National Institute for Health and Care Research (NIHR) Biomedical Research Centre (BRC) at the South London and Maudsley NHS Foundation Trust (SLaM) and King's College London (KCL).*

### Conflicts of interest

*H.H. is the chief investigator for an NIHR HTA-funded feasibility study testing olanzapine in young patients with anorexia nervosa and the principal investigator of a COMPASS Pathways-funded and sponsored proof-of-concept study testing psilocybin in anorexia nervosa. H.H., J.L.K. and J.T. report funding from the UK Medical Research Council (MRC) for a randomized controlled feasibility trial to test oral ketamine in people with anorexia nervosa and depression, which will start in 2025. K.T. declares no conflicts of interest.*

## REFERENCES AND RECOMMENDED READING

Papers of particular interest, published within the annual period of review, have been highlighted as:

▪ of special interest
▪▪ of outstanding interest
